# Building Blocks of Others' Understanding: A Perspective Shift in Investigating Social-Communicative Deficit in Autism

**DOI:** 10.3389/fnhum.2016.00144

**Published:** 2016-04-12

**Authors:** Luca Ronconi, Massimo Molteni, Luca Casartelli

**Affiliations:** ^1^Developmental and Cognitive Neuroscience Lab, Department of General Psychology, University of PaduaPadua, Italy; ^2^Child Psychopathology Unit, Scientific Institute IRCCS Eugenio MedeaBosisio Parini, Italy; ^3^Developmental Psychopathology Unit, Vita-Salute San Raffaele UniversityMilan, Italy

**Keywords:** joint attention, social cognition, visual perception, auditory perception, visual attention, multisensory integration

## Social cognition in autism and the need for a perspective shift

Autism Spectrum Disorder (ASD) is a neurodevelopmental lifelong condition affecting over 1% of the population and characterized by significant impairments in social communication and interaction, as well as by the presence of restricted and repetitive patterns of behavior, interests and activities (American Psychiatric Association, 2013). With the recent introduction of DSM-V, hyper- or hyporeactivity to sensory input or unusual responses to sensory aspects of the environment are also acknowledged among the possible behavioral manifestations of ASD (American Psychiatric Association, 2013). Pharmacological therapies can reduce comorbid symptoms, but do not directly improve social-communicative deficits (Lai et al., 2014). Thus, early detection/(reh)abilitation programs that improve symptoms and social functioning are crucial (Rogers et al., 2014; Wass and Porayska-Pomsta, 2014). Among other explanations (Lai et al., 2014), the impaired “social brain” hypothesis and the theory of mind (ToM) deficit have been considered for decades among the most reasonable explanations for social interactions difficulties in ASD (Baron-Cohen et al., 1985; Pelphrey et al., 2004; Blakemore, 2010; Burnett et al., 2011; Gotts et al., 2012; Vissers et al., 2012); however, both the presence of heterogeneous experimental protocols (Boucher, 2012) and controversial neuroimaging findings (Mitchell, 2008; Schurz et al., 2014) suggests a methodological and conceptual shift (Schaafsma et al., 2015). Going beyond the difficulties in defining the concepts of ToM and “social brain” in operational terms (Gallese, 2007; Casartelli and Molteni, 2014; Casartelli and Chiamulera, 2016), in this opinion article we propose a perspective shift. This shift is based on the idea that core social-communicative deficits of ASD may be more efficiently tackled starting from the comprehension of anomalies in basic functions of different sensory and cognitive domains: specifically, visual/auditory perception, multisensory integration and attention (for a similar approach in the motor domain, see Casartelli et al., 2016). The developmental trajectories of these basic functions are supposed to play a key role in the (a)typical brain development, and we consider them as the “building blocks” of social skills development.

To some extent, this idea is not completely new even if it has not been tackled *per se*, at least in the context of ASD. The classic neuroconstructivist approach, indeed, recognizes the presence of innate (or very early), not detailed and not domain-specific biological constraints on (a)typical development (Karmiloff-Smith, 1998). A study by Pellicano (2010) is in line with this idea. She investigated the longitudinal relationship among the ToM and both executive functions (EF) and central coherence, which are hypothesized to be anomalous in ASD. Her findings provide important evidence that executive functions (i.e., domain general skills) unidirectionally predict the developmental trajectory of ToM skills, raising the question of potential confounds and biases in the definition of concepts as ToM or “social brain.”

Here, we consider the growing body of experimental studies (not pretending to be exhaustive) that *directly* demonstrate a link between basic non-social and basic social functions in ASD. With *direct* demonstration, we intend a direct investigation—in the same study and on the same individuals—of profiles/performances in non-social and social tasks. We focus on different and paradigmatic domains: visual and auditory perception, multisensory integration, visual attention.

## Basic sensory and cognitive markers of social-communicative impairments in autism

Recent studies corroborate the idea that difficulties in visual processing of social information (e.g., faces, biological motion) are part of a pervasive processing atypicality in ASD that may affect how these individuals process social and non-social stimuli in the same way. Ewing et al. (2013) asked whether face-processing difficulties in autism are disproportionate to difficulties with other complex non-face stimuli. To demonstrate such a selectivity, according to the authors, there must be a significant interaction between participants' group (ASD vs. non-ASD) and stimulus category (faces vs. non-faces). Ewing et al. (2013) results showed no evidence for face-selective processing difficulties in the ASD group, thus raising question about the widespread view (Dawson et al., 2005; Webb et al., 2011) that faces pose a special perceptual problem for individuals with autism. The hypothesis of a pervasive processing deficit has been tested also by Vlamings et al. (2010). They measured electroencephalography (EEG) in response to nonsocial (gratings) and social (facial expressions) stimuli, focusing on two event-related potentials (ERPs) that are sensitive to the spatial frequency content of both stimulus types: (i) the P1, which reflects activity of early striate/extrastriate visual areas (Di Russo et al., 2002); (ii) the N170, a face-specific component, which reflects a later processing stage that includes the fusiform face area (Henson et al., 2003). The results showed an enhanced neural activity to high (HSF) as compared to low (LSF) spatial frequency in the ASD group for nonsocial stimuli (gratings) in the early stages of visual processing (P1). Importantly, the same bias toward HSF processing was evident in the extraction of emotional expressions, contrarily to the control group that relied on LSF. Thus, the study of Vlamings et al. (2010) shows that processing of emotional content in the early course of ASD is present, but is atypical as it is based on HSF (i.e., detail processing). This early HSF/LSF sensitivity imbalance, according to the authors, is likely to impact on the development of the adult face processing network. A further study by Kroger et al. (2014) employed again EEG to clarify which components in the temporal sequence of neural processes are disturbed during biological (social) and scrambled (non-social) motion perception in ASD. Their results showed a reduced amplitude of the P1 in both biological and scrambled motion processing in adolescent with ASD. The P1 reflects elementary stimulus features processing, as well as early detection of simple motion (Krakowski et al., 2011). The authors found also that this abnormality at the P1 level may partly explain reduced subsequent activation in the biological motion specific components (N200 and P400).

Although less extensively investigated compared to the visual domain, abnormalities in the auditory perceptual domain in ASD are also reported (O'Connor, 2012; Kujala et al., 2013). In particular, neurophysiological studies employing EEG or magnetoencephalography (MEG) showed that abnormalities in auditory perception: (i) seem to originate from the early stages of neural processing (e.g., auditory P1/P1m/M50, N1/N1m/M100 and Mismatch Negativity/Magnetic Mismatch Field; Gage et al., 2003; Oram-Cardy et al., 2005a; Roberts et al., 2010, 2011; Edgar et al., 2014), (ii) seem to partly depend on the pre-stimulus oscillatory activity (Edgar et al., 2015); (iii) seem to be present for both speech and non-speech sounds (Oram Cardy et al., 2005b; but see Čeponienė et al., 2003). Correlational studies suggest also a relationship between these early electrophysiological abnormalities in the auditory domain and behavioral measures of emotion recognition in ASD (Lerner et al., 2013; Demopoulos et al., 2015). However, few studies have directly tested the performance of individuals with ASD to both low- (non-linguistic) and high- (linguistic) level stimuli. Järvinen-Pasley and Heaton (2007) tested if there was a different pitch sensitivity in ASD for non-speech relative to speech stimuli. A same/different discrimination task that comprised three conditions of increasing complexity was used. The control group, as hypothesized, exhibited a lower pitch sensitivity in the conditions that required the discrimination of speech. This result is consistent with the idea that attention to both content and intonation cues during speech processing would limit the processing capacity/resources available for low-level pitch analysis. On the contrary, children with ASD exhibited the same pitch sensitivity across all experimental conditions. These findings –confirmed also in subsequent studies (Järvinen-Pasley et al., 2008a,b)—suggest a reduced domain specificity of the auditory processing in ASD, i.e., a similar sensitivity to pitch across different stimulus domains.

A further domain of interest for the perspective shift proposed here is multisensory integration (MSI). MSI allows us to know what information belong together and what information should be segregated, leading to different behavioral benefits, including ameliorations in speech comprehension (Stevenson et al., 2014a). For example, recent evidence show that the cortical entrainment to continuous auditory speech is enhanced when visual speech that shares the same timing is presented, and this effect seems to depend on different cortical generators relative to those that are active during unimodal speech presentation (Crosse et al., 2015). Many factors influence how we integrate sensory signals across the different modalities. One of the strongest is the ability to perceive temporal relationships between the sensory inputs. Impairments in temporal processing and MSI are well documented in ASD (Brock et al., 2002; Foss-Feig et al., 2010; Kwakye et al., 2011). In a recent study, Stevenson et al. (2014b) directly tested the hypothesis that alterations in multisensory temporal processing may be related to deficits in audiovisual integration of speech in individuals with ASD. A temporal binding window (TBW)—that measured the time within which multisensory inputs are highly likely to be perceptually bound—was estimated in different multisensory tasks with audiovisual stimuli that ranged from simple flash/beep pair to complex speech. The main result of Stevenson et al. (2014b) showed that individuals with ASD had larger TBW specifically for speech stimuli. Importantly, the authors found that the strength of perceptual binding of audiovisual speech observed in individuals with ASD was strongly related to their low-level multisensory temporal processing abilities. The poorer an individual's temporal acuity across vision and audition (i.e., the larger their TBW), even with simple flashes and beeps, the weaker their ability to bind auditory and visual speech information. The study of Stevenson et al. (2014b) is highly informative because it is the first to establish a clear link between aspects of multisensory processing and the higher-order domain of speech processing.

Finally, an interesting domain for our perspective shift is visual attention. It can be considered as the mechanism through which we select important information in the visual environment, thereby determining what we experience and respond to. An early-onset disorder that interferes with the typical attention development trajectory may have wide effects on social-communicative development. Numerous visual attention deficits have been associated to ASD (Ames and Fletcher-Watson, 2010), affecting the abilities to rapidly orient (Keehn et al., 2013) and to re-orient or disengage (Sacrey et al., 2014) the focus of attention, but involving also the ability to adjust its size (Mann and Walker, 2003; Ronconi et al., 2012, 2013, 2016). In particular, evidence from infancy, childhood, and adulthood show that disengagement is impaired in ASD and its broader phenotype (Ronconi et al., 2014; Sacrey et al., 2014). Moreover, prospective studies of visual disengagement during the first years of life suggest that impairments in this function are evident by 12 months of age in at-risk infants (i.e., siblings of older children with ASD who are at higher risk of developing the condition; Bolton et al., 1998) who later receive an ASD diagnosis (Elsabbagh et al., 2013; Sacrey et al., 2013). Despite the diffuse idea that visual disengagement is necessary for normal social development (Dawson and Lewy, 1989), particularly for the development of joint attention, only recently studies have directly tested the link between basic non-social and basic social visual attention. Schietecatte et al. (2012) investigated attentional disengagement abilities through a gap-overlap paradigm (Saslow, 1967) in a group of children with ASD in relation to their joint attention skills. Their results indicated that children who were rapid to disengage their attention showed a higher propensity to initiate joint attention. This evidence is consistent with previous studies in typically developing infants showing that the degree of which attention is captured by changes in the visual environment directly influence joint attention abilities (Butterworth and Grover, 1990; Butterworth and Jarrett, 1991).

A fundamental point that will need to be tackled in future studies is whether the impairments in non-social mechanisms are causal factors or simply associated dysfunctions in the impaired processing of social stimuli. One possible way to clarify this issue is to undertake longitudinal studies of infants at risk for ASD. In a recent study by Bedford et al. (2014), the authors studied the influence of both non-social (disengagement) and social (gaze following) attentional functions in infants at risk. Their results showed that both mechanisms significantly predict later ASD diagnosis, raising the question about which is the primary (and more early detectable) deficit in the pathophysiology of ASD. Future longitudinal studies in high-risk infants should be designed also to evaluate which mechanism (social vs. non-social) derails first from the typical developmental trajectory.

## Concluding remarks: Implications for current autism research

These works, according to the perspective shift proposed here, show how the study of social difficulties in ASD may take advantage of a more domain general approach. Approaching the study of ASD with this perspective shift could be promising for a number of important aspects. First, impairments or anomalies in basic functions can be considered as markers of ASD that could be useful to improve early detection and to set (reh)abilitative protocols before the onset of unequivocal behavioral symptoms. In addition, this perspective shift would support translational research, as these basic non-social functions are easier to investigate in animal models and easier to map onto specific genetic/epigenetic factors compared to complex social phenotypes. Finally, this perspective shift may be useful to better characterize the ontogeny of complex concepts as “social brain” or ToM, deconstructing them in more elementary components.

## Author contributions

All the three authors developed the idea behind this work. LR and LC drafted the manuscript and MM revised it critically. All the authors gave approval for the final version.

## Funding

This work was supported by a grant from the Italian Ministry of Health (Ricerca Corrente 2014-2015) to LC.

### Conflict of interest statement

The authors declare that the research was conducted in the absence of any commercial or financial relationships that could be construed as a potential conflict of interest. The reviewer [CD] and handling Editor declared their shared affiliation, and the handling Editor states that the process nevertheless met the standards of a fair and objective review.
